# Patient-rated level of discomfort during assessment with point-of-care ultrasonography

**DOI:** 10.1186/1757-7241-23-S1-A17

**Published:** 2015-07-16

**Authors:** Christian B Laursen, Erik Sloth, Annmarie T Lassen, Jess Lambrechtsen, Daniel P Henriksen, Finn Rasmussen

**Affiliations:** 1 Department of Respiratory Medicine, OUH Odense University Hospital, Odense, Denmark; 2 Department of Anesthesia and Intensive Care, Aarhus University Hospital, Skejby, Denmark; 3 Department of Emergency Medicine, OUH Odense University Hospital, Odense, Denmark; 4 Department of Medicine, OUH Odense University Hospital, Svendborg, Denmark; 5 Institute of Clinical Research, University of Southern Denmark, Odense, Denmark; 6 Department of Allergy and Respiratory Medicine, Near East University Hospital, Nicosia, North Cyprus, Mersin 10, Turkey

## Introduction

This study aimed to assess the patient-rated level of discomfort during point-of-care ultrasonography of the heart, lungs, and deep veins in a population of patients admitted to an emergency department with respiratory symptoms and to what extent the patients would accept being assessed by the use of point-of-care ultrasonography if they had to be examined for possible disease.

## Methods

A questionnaire-based observational study was conducted in an emergency department. Inclusion criteria were one or more of the following: respiratory rate > 20/minute, oxygen saturation < 95%, oxygen therapy initiated, dyspnoea, cough or chest pain. Patients were examined by the use of point-of-care ultrasonography of the heart, lungs, and deep veins. Patient-rated level of discomfort and acceptance were assessed using a standardised questionnaire.

## Results

A total of 1,130 patients were assessed for eligibility, of which 299 (26.5%) patients were included. 26 patients was not able to fill out the questionnaire and 2 patients withdrew informed consent, leaving 271 patients available for study analysis. The median duration of the sonographic examinations was 12 minutes (IQR 11-13, range 9-23). The median patient-rated level of discomfort for all three types of sonography was 1 (IQR 1-1, range 1-8) on a scale from 1 to 10. All but one patient (99.6% (95% CI: 98.9-100%)), would accept being examined by the use of point-of-care ultrasonography as a part of routine emergency department diagnostics.

## Conclusion

The patient-rated level of discomfort during point-of-care ultrasonography of the heart, lungs, and deep veins is very low and the vast majority of patients would accept being assessed by the use of point-of-care ultrasonography if the patients once again had to be examined for possible disease.

